# Faecal Viromes of Three Sympatric Penguins on Ardley Island, Antarctica: A Transcriptomic Study Showing Limited Host-Species Effect

**DOI:** 10.3390/v18101138

**Published:** 2026-10-14

**Authors:** Ruifeng Xu, Minhao Gao, Wenting Mao, Jian Xiao, Yang Liu, Jun Ni, Yi Han, Yufei Dai, Haizhou Liu, Fei Deng, Liulin Chen, Wei Wang

**Affiliations:** 1 Wuhan Institute of Virology, Chinese Academy of Sciences, Wuhan 430071, China; xurf@wh.iov.cn (R.X.);; 2 Antarctic Great Wall Ecology National Observation and Research Station, Polar Research Institute of China, Ministry of Natural Resources, Shanghai 200136, China; 3 University of the Chinese Academy of Sciences, Beijing 101408, China; 4 Chongqing Key Laboratory of Highly Pathogenic Microbes, Chongqing Research Center for Disease Prevention and Public Health, Chongqing Center for Disease Control and Prevention (Chongqing Academy of Preventive Medicine), Chongqing 400707, China; 5 Polar Research Institute of China, Shanghai 200136, China

**Keywords:** pygoscelis, phylogenetic analysis, virome, deltacoronavirus, astrovirus, picornaviridae, Antarctic viral ecology

## Abstract

During the 41st Chinese Antarctic Expedition (2024–2025), fresh faecal samples were collected from sympatric Adélie, chinstrap, and gentoo penguins on Ardley Island. The faecal viromes were systematically profiled via transcriptomic sequencing coupled with viral contig assembly. Thirty-two known viral families were annotated, yet over half of viral reads remained unclassified, indicating substantial uncharacterised viral diversity in Antarctic penguin guts. No significant interspecific differences in viral abundance or alpha-diversity were detected, and host-species exerted only a weak effect on viral community structure. While H5N1 highly pathogenic avian influenza and Newcastle disease virus were absent, several potential pathogens, including deltacoronaviruses, novel astroviruses, and picornaviruses, were recovered. This work establishes baseline virome data for Antarctic pygoscelid penguins and provides valuable insights for long-term pathogen surveillance of polar wildlife and assessments of viral spillover risk.

## 1. Introduction

Ardley Island, located in the Fildes Peninsula region of the western Antarctic Peninsula and adjacent to King George Island of the South Shetland Islands, represents one of the largest ice-free areas in maritime Antarctica [1]. Compared with continental Antarctica, its relatively mild temperatures and greater precipitation sustain diverse nesting seabird populations and extensive vegetation. Three sympatric *Pygoscelis* penguin species (Adélie, gentoo, and chinstrap) breed on the island. Long-term host–virus co-evolution has enabled these penguins to maintain stable reservoirs of low-pathogenicity viruses, including avian influenza viruses [2,3], astroviruses [4], paramyxoviruses [5,6], circoviruses [7], adenoviruses [8], and polyomaviruses [9]. Despite growing viral detection reports [10], systematic comparisons of virome composition and interspecific viral diversity across among these sympatric species remain lacking, a limitation imposed by small sample sizes and incomplete viral coverage. To fill this knowledge gap, fresh faecal samples were collected from the three *Pygoscelis* species between December 2024 and April 2025. Using transcriptomic sequencing, viral contig assembly, and taxonomic annotation, we characterised viral communities, interspecific viral diversity, and the distribution of high-risk pathogens, thereby establishing baseline virome datasets for long-term wildlife surveillance and ecological risk assessment in polar ecosystems.

## 2. Materials and Methods

### 2.1. Sample Collection and Host Species Identification

Fresh faecal samples of Adélie, chinstrap, and gentoo penguins were collected on Ardley Island between December 2024 and April 2025. Fresh faecal samples were defined as freshly deposited droppings with moist surfaces, no signs of freeze–thaw cycles, and no visible fungal or bacterial overgrowth. Samples were collected within approximately 24 h after defecation based on field visual inspection. The samples were immersed in RNAlater (Thermo Fisher Scientific, Waltham, MA, USA) and stored at −80 °C prior to laboratory analysis. Host species identification was confirmed by integrating penguin morphological characteristics and mitochondrial cytochrome c oxidase subunit I (*COI*) gene sequence alignment.

### 2.2. Total RNA Extraction and High-Throughput Library Construction

Total RNA was isolated from individual faecal samples using the spin-column-based Faecal Total RNA Fast Extraction Kit (Bioteke Corporation, Wuxi, China) according to the manufacturer’s protocol. Briefly, faecal material was homogenised and lysed in lysis buffer DL supplemented with 4% β-mercaptoethanol. After protein precipitation, supernatants were mixed with 70% ethanol and loaded onto RNase-free spin-columns. Serial washing steps with protein-removal reagent RE and wash buffer RW were performed to remove protein and inhibitory contaminants. Nucleic acids were finally eluted in pre-heated RNase-free water. After elution, the integrity, purity, and concentration of RNA were determined with an Agilent 2100 Bioanalyzer (Agilent Technologies, Santa Clara, CA, USA) and NanoDrop (Thermo Fisher Scientific, Wilmington, DE, USA) before library construction for nucleic-acid quality evaluation. mRNA was purified from total RNA using poly-T oligo-attached magnetic beads. After fragmentation, the first strand of cDNA was synthesised using random hexamer primers followed by second-strand cDNA synthesis. The library was ready after end repair, A-tailing, adapter ligation, size selection, amplification, and purification. After library quality control, different libraries were pooled based on the effective concentration and targeted data amount. A 5′-end of each library was phosphorylated and cyclized. Subsequently, loop amplification was performed to generate DNA nanoballs. These DNA nanoballs were loaded onto a flow cell. Paired-end (PE) 150 bp sequencing was performed on the DNBSEQ-T7 platform (BGI, Shenzhen, China) using combinatorial probe–anchor synthesis (cPAS) sequencing technology. Library sequencing was conducted by Novogene, Beijing, China.

### 2.3. Quality Control and Sequence Assembly

Quality control and assembly of the sequencing data were performed as described previously [11]. Raw sequencing reads were first evaluated with FastQC (v0.12.1). Trimmomatic (v0.39) was then applied to remove low-quality reads and adapter sequences to generate cleaned reads. Cleaned reads were subjected to de novo assembly using MEGAHIT (v1.2.9) to generate contigs. Contigs assembled from all groups were subjected to homology searches against the NCBI GenBank non-redundant protein database (nr) and nucleotide database (nt; December 2025 release). DIAMOND-BLASTX (v2.1.11.165) was used for protein-level searches, and BLASTN (v2.16.0) for nucleotide-level searches. The E-value cutoff was set to 10^−10^ for both searches; all other parameters were kept as default. Cleaned reads were mapped against assembled viral contigs using Bowtie2 (v2.5.4). The resulting alignment files were sorted and indexed with SAMtools (v1.16.1). CoverM (v0.7.0; default parameters) was used for abundance quantification of virus-annotated contigs using the pre-processed BAM files as input. Read counts were normalised to transcripts per million (TPM), which was calculated exclusively over virus-annotated contigs to represent relative viral abundance within the viral fraction of each sample. All viral nucleotide sequences generated in this study are publicly available in the Science Data Bank (ScienceDB): DOI https://doi.org/10.57760/sciencedb.0104t, CSTR 31253.11.sciencedb.0104t.

### 2.4. Viral Community Diversity Analysis

Viral community analyses were conducted at the family taxonomic level. Alpha diversity indices, including richness and Shannon index [12], were calculated for each sample. Differences among host species groups were evaluated using Kruskal–Wallis tests. Beta diversity was assessed based on Bray–Curtis dissimilarity matrices, and host species effects were tested using PERMANOVA with 999 permutations. Ordination analyses were performed using non-metric multidimensional scaling (NMDS) and principal coordinate analysis (PCoA). Hierarchical clustering was conducted using Wards method. All diversity and abundance visualisations were generated using pheatmap (v1.0.12) and ggplot2 (v3.4.4).

### 2.5. Phylogenetic Analysis

Phylogenetic reconstructions were carried out based on deltacoronavirus genomic sequences and *RdRp* gene sequences, astrovirus *RdRp* gene sequences and RdRp, ORF2 capsid protein sequences, *Picornaviridae* RdRp protein sequences, and *Caliciviridae* RdRp protein sequences obtained in this study, alongside homologous reference sequences retrieved from the NCBI database. Sequences were aligned using MAFFT (v7.471), and the optimal substitution models were selected by the built-in ModelFinder module of IQ-TREE (v1.6.8). Maximum-likelihood (ML) phylogenetic trees were constructed using IQ-TREE (v1.6.8) with 1000 bootstrap replicates. Tree visualisation and refinement were conducted using FigTree (v1.4.4). For pairwise identity heatmaps, gap-free pairwise similarity values were calculated separately on trimmed MAFFT alignments of target gene regions (RdRp nucleotides and capsid amino acids for astroviruses). Independent hierarchical clustering was performed for each gene matrix, and dual-panel heatmaps illustrating sequence identity were generated using the R package pheatmap (v1.0.13). The gene fragments used for phylogenetic reconstruction were extracted from assembled viral contigs. The length of each fragment used for phylogenetic analysis and tree reconstruction is available in Table A1.

## 3. Results

### 3.1. Sample Collection and Host Species Composition

**Figure 1:**
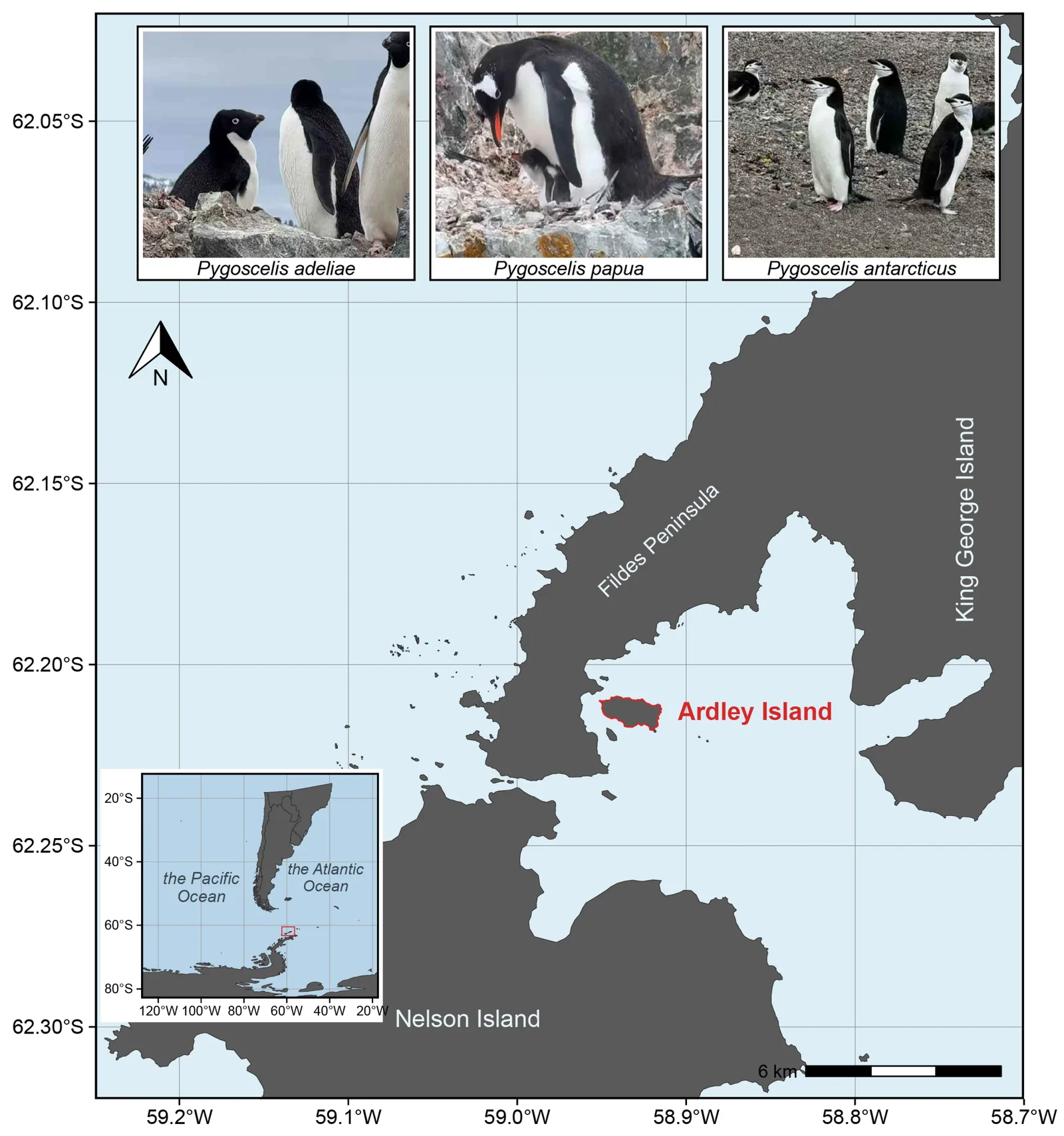
Sampling map and images of three penguin species. Ardley Island (red label) in the South Shetland Islands was selected as the sampling location. The inset map shows the overall geographic position of the Antarctic Peninsula. The three pictures at the top show *Pygoscelis adeliae*, *Pygoscelis papua* and *Pygoscelis antarcticus*, respectively, which are the host species for faecal sample collection in the present work. Map elements include latitude and longitude grids, north indicator, and a 6 km distance scale bar.

Sample collection was carried out on Ardley Island adjacent to Chinese Great Wall Station (Fildes Peninsula, Antarctica) between December 2024 and February 2025. Fresh faecal samples collected from chinstrap, Adélie, and gentoo penguins were subjected to total nucleic acid extraction, library preparation and transcriptomic sequencing (Figure 1). In total, 32 qualified faecal transcriptome samples were obtained. Host species assignment was confirmed by combining field morphological observation with mitochondrial COI gene sequence alignment. This sample set consisted of 14 Adélie penguin, 9 chinstrap penguin, and 9 gentoo penguin specimens.

### 3.2. Composition and Diversity of Viral Communities in Faecal Samples

**Figure 2:**
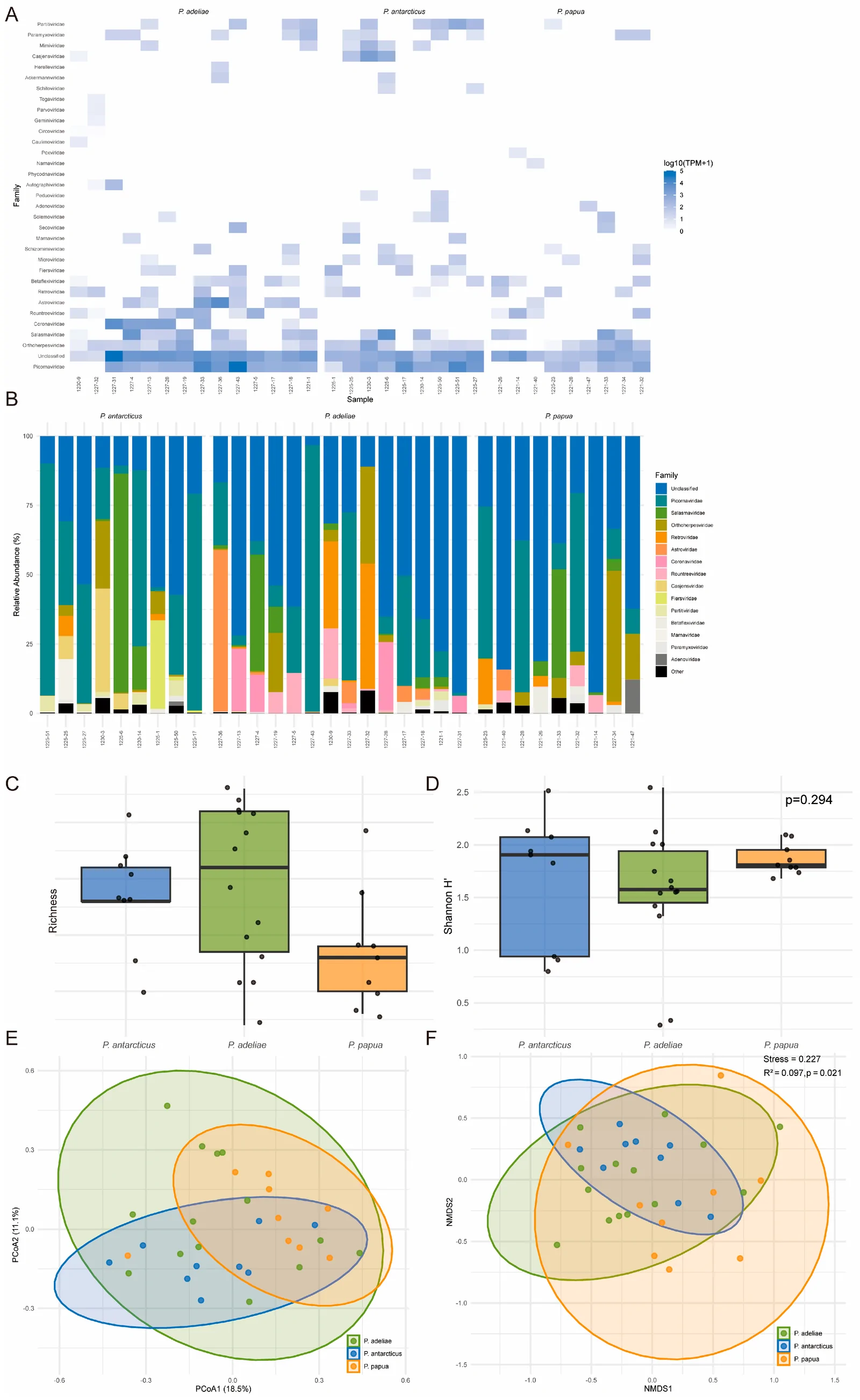
Composition and diversity of viral communities in faecal samples of three sympatric Pygoscelis penguin species. (**A**) Heatmap of log_10_-transformed read counts at the family level across individual samples. The colour gradient represents relative read abundance. Viral family names are indicated in the left legend. (**B**) Stacked bar chart displaying the relative abundance of viral families in each sample. The *y*-axis represents the proportion of viral reads assigned to each family; the *x*-axis indicates sample ID; colours correspond to viral families as shown in the legend. (**C**,**D**) Alpha diversity of viral communities assessed by species richness (**C**) and Shannon–Wiener index (**D**) (Kruskal–Wallis test; richness: *p* = 0.085; Shannon: *p* = 0.294). Box colors correspond to three penguin host species: *P. antarcticus* (blue), *P. adeliae* (green), and *P. papua* (orange). (**E**) PCoA and (**F**) NMDS ordinations based on Bray–Curtis dissimilarity, showing weak host species clustering. PERMANOVA result: R^2^ = 0.097; *p* = 0.021. Coloured ellipses represent 95% confidence intervals for each host species group.

**Figure A1:**
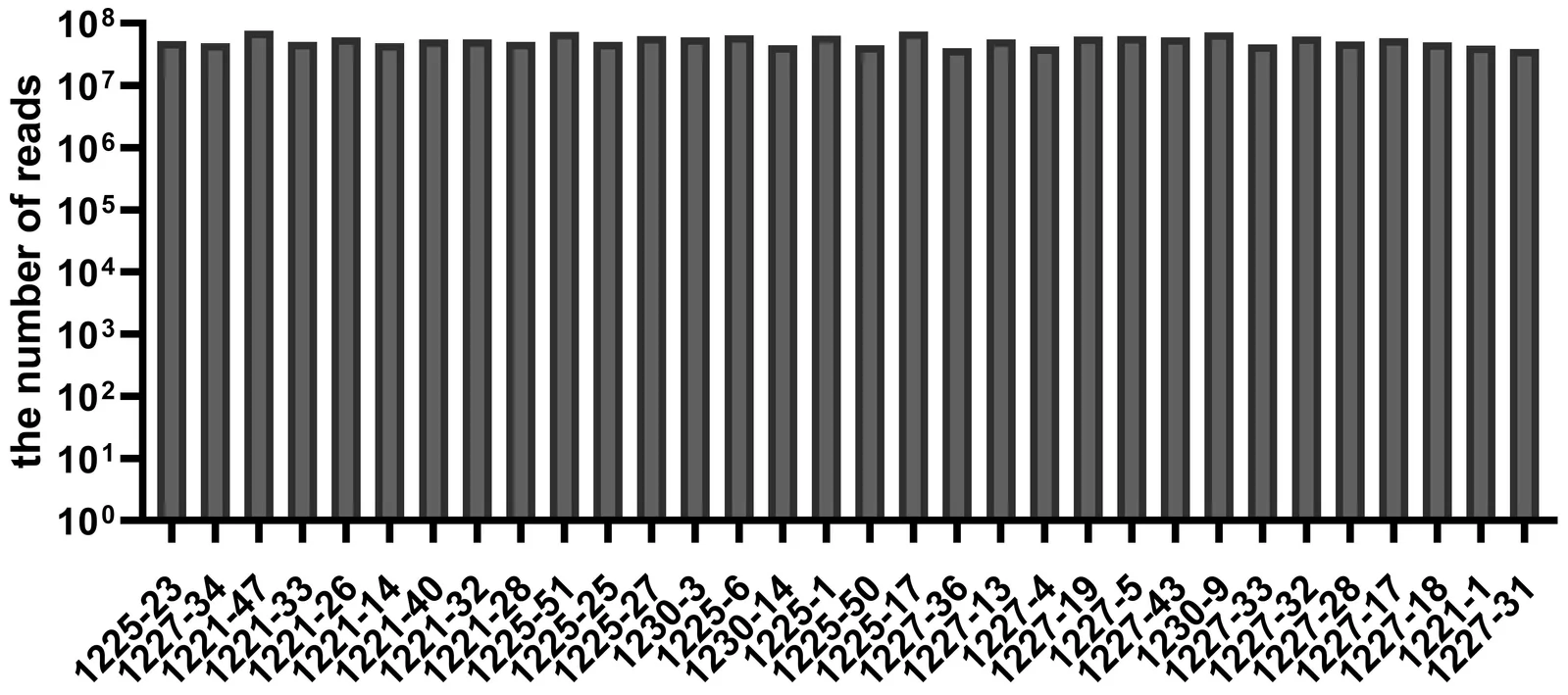
Clean read counts for the 32 faecal transcriptome samples of Antarctic penguins. The *y*-axis presents the number of clean reads on a log10 scale, and sample identifiers are labelled on the *x*-axis. All samples yielded sufficient sequencing data for subsequent virome investigation.

A total of 32 faecal transcriptome libraries were sequenced. Following quality trimming, the number of clean reads per sample ranged from 38.33 million to 75.50 million, with a mean of 54.77 million clean reads across all samples. Sequencing depth was adequate for subsequent virome characterisation (Figure A1). Raw reads were subjected to quality control filtering, de novo contig assembly, and taxonomic annotation. In total, 32 known viral families were identified in the dataset. After removing bacteriophages and retroviruses, 23 known eukaryotic viral species remained for downstream analyses, which included profiling of viral community composition profiling, diversity assessment, and characterisation of predominant viral pathogens (Figure 2A).

Notably, unclassified sequences accounted for more than half of all viral reads across the three host species, indicating that Antarctic penguin gut ecosystems harbour substantial undescribed viral diversity. Annotated viruses included both RNA and DNA groups. Major RNA viral taxa included *Picornaviridae*, *Coronaviridae*, *Astroviridae*, *Paramyxoviridae* and *Rountreeviridae*. *Picornaviridae* was more abundant in chinstrap and Adélie penguins relative to gentoo penguins, while *Coronaviridae* was highly enriched specifically in Adélie penguins. Several RNA viruses derived from algae and plant were also detected, which were considered to originate from the penguins’ dietary intake. Dominant DNA viruses included *Herpesviridae*, *Adenoviridae* and *Poxviridae*, together with giant DNA viruses (*Pandoraviridae*, *Mimiviridae*, *Schizomimiviridae*), *Herpesviridae* exhibited preferential accumulation in chinstrap penguins, whereas giant DNA viruses were present at higher abundance in gentoo penguins. Algal and plant DNA viruses (*Phycodnaviridae*, *Caulimoviridae*) were also identified as food-borne contaminants (Figure 2B). Critically, no H5N1 HPAIV or Newcastle disease virus was detected in all faecal samples.

Viral alpha diversity was further characterised based on species richness and Shannon’s diversity index. Kruskal–Wallis tests revealed no significant interspecific differences in viral species richness (*p* = 0.085) or Shannon diversity (*p* = 0.294) among the three penguin species (Figure 2C,D). Beta diversity, assessed via Bray–Curtis dissimilarity, showed that the first two PCoA axes cumulatively explained 29.5% of total variation (PCoA1: 18.5%; PCoA2: 11.1%), consistent with the NMDS ordination results (Stress = 0.227; Figure 2E,F). Although PERMANOVA detected a statistically significant effect of host species on viral community structure (R^2^ = 0.097, *p* = 0.021), the low R^2^ value indicates weak host filtering, suggesting that host species does not drive the formation of species-specific viromes. Most compositional divergence among samples stems from host-level characteristics, dietary differences, environmental exposures, and stochastic processes.

### 3.3. Genomic and Phylogenetic Characteristics of Adélie Penguin-Derived Deltacoronavirus

**Figure 3:**
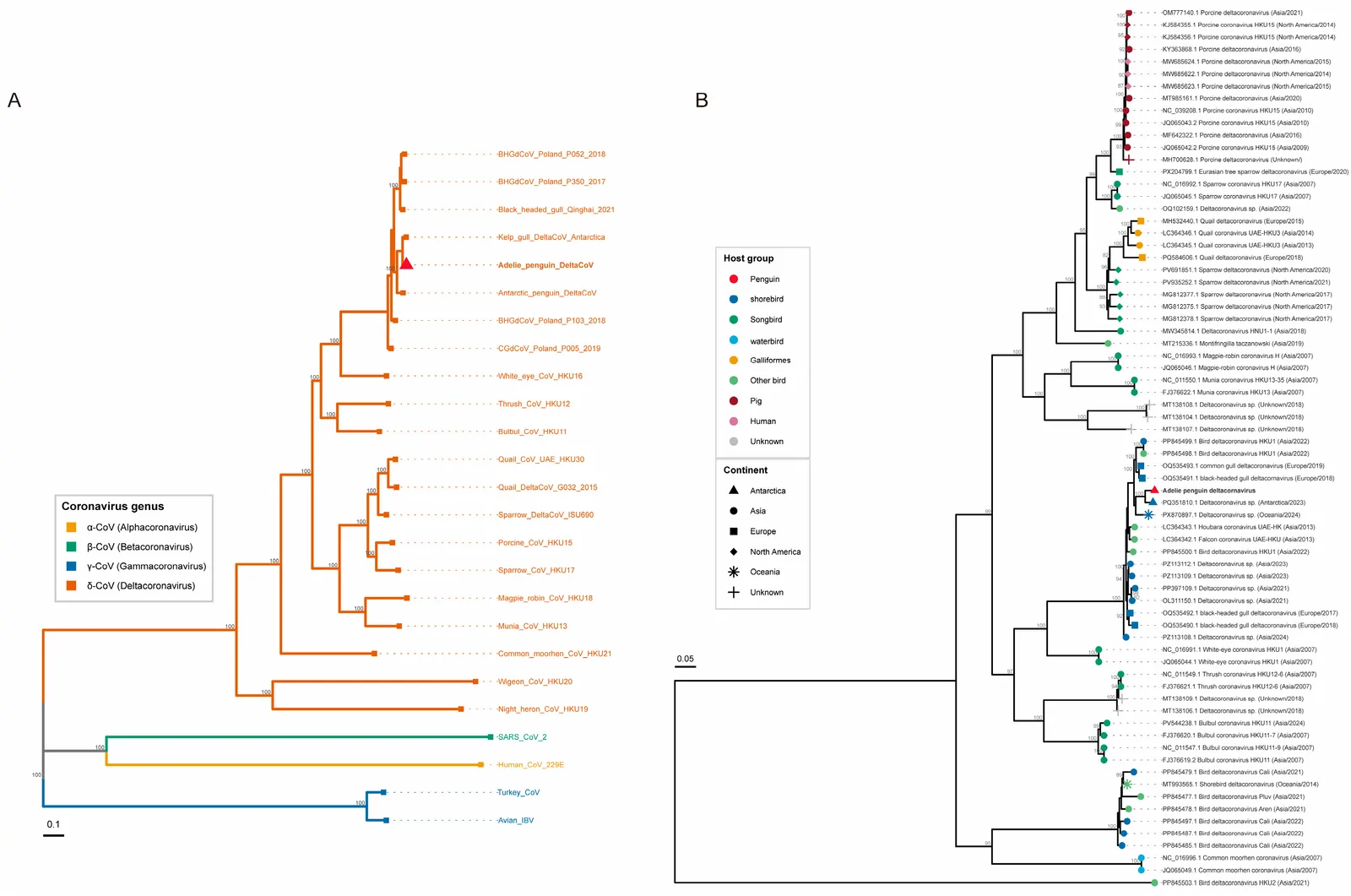
Phylogenetic placement of deltacoronaviruses from Antarctic penguins. (**A**) Maximum-likelihood tree based on trimmed core genomic regions of deltacoronaviruses (23,506 nt). Multiple-sequence alignment was performed using MAFFT. ML tree built by IQ-TREE with 1000 bootstrap replicates. Branches are colour-coded to denote distinct coronavirus genera and subgenera. Nodes displaying bootstrap support values ≥ 80 are explicitly labelled, and the scale bar corresponds to pairwise genetic distance. Strains recovered in the present study are highlighted with red triangular symbols. (**B**) Midpoint-rooted phylogenetic tree inferred from RdRp gene sequences (2772 nt). Multiple-sequence alignment was performed using MAFFT. ML tree built by IQ-TREE with 1000 bootstrap replicates. Terminal tips are coloured according to host taxonomy and shaped by geographic continent of strain origin. Red triangles mark deltacoronavirus isolates characterised in this study; bootstrap values ≥ 80 is annotated at respective nodal positions.

A near-complete deltacoronavirus genome (26,196 nt) was successfully assembled from a single Adélie penguin faecal sample (1227-13). Even read-depth distribution across the contig and high genome-wide coverage corroborated the reliability of this genomic assembly. This virus was formally designated “Adélie penguin deltacoronavirus”. Maximum-likelihood phylogenetic trees were constructed based on these trimmed genomic sequences as well as RNA-dependent RNA polymerase (*RdRp*) replicase gene sequences. Phylogenetic analysis revealed that this strain falls within the subgenus Buldecovirus of the family *Coronaviridae*, and clusters with a deltacoronavirus recovered from Antarctic sheathbills in 2023, as well as a 2014 deltacoronavirus sequence originally identified in gentoo penguins (Figure 3A). Multiple genomic comparisons among deltacoronavirus sequences revealed that this isolate shared the highest nucleotide identity, of 96.23%, with the 2023 Antarctic sheathbill deltacoronavirus (GenBank accession: PQ351810.1). RdRp-based phylogenetic analysis indicated substantial genetic differentiation between polar deltacoronavirus isolates and those circulating among avian populations on other continents (Figure 3B). These findings suggest sustained cross-species transmission of deltacoronaviruses among Antarctic seabirds and the emergence of a distinct Antarctic deltacoronavirus lineage.

### 3.4. Genomic and Phylogenetic Characteristics of Penguin Astrovirus

**Figure 4:**
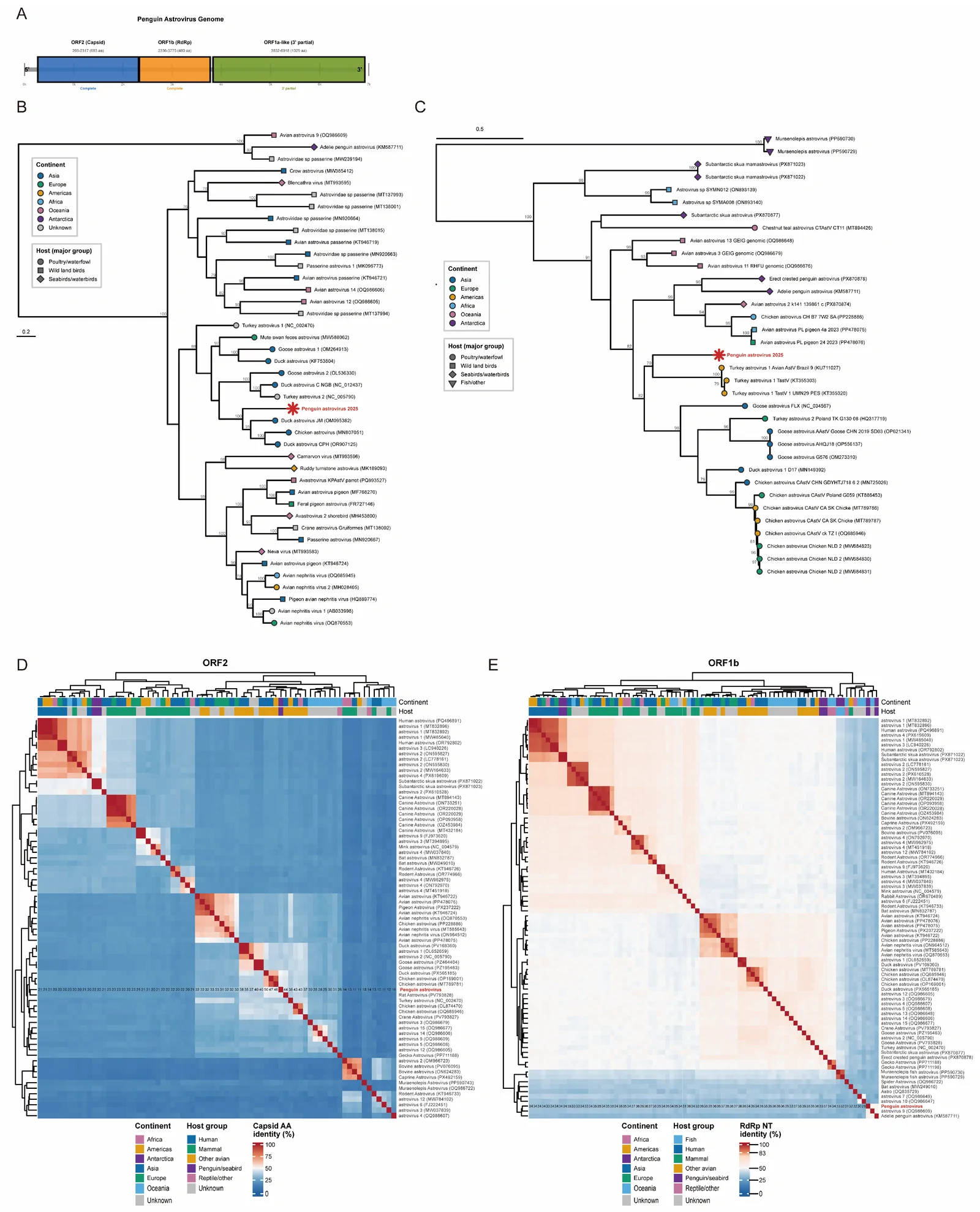
Genome organisation and phylogenetic characterisation of Penguin astrovirus 2025. (**A**) The genome encodes a protease (ORF1a) and RNA-dependent RNA polymerase (ORF1b), as well as the capsid protein (ORF2). (**B**,**C**) Maximum-likelihood phylogenetic trees of Penguin astrovirus 2025 based on amino acid sequences of RdRp ((**B**); 480aa) and ORF2 ((**C**); 683aa). Multiple-sequence alignment was performed using MAFFT. ML tree built by IQ-TREE with 1000 bootstrap replicates. Trees are colour-coded by continent of detection and shaped by host major group. The strains identified in this study are indicated by red asterisk. Scale bars represent amino acid substitutions per site. (**D**,**E**) Pairwise sequence identity heatmaps of Penguin astrovirus 2025 based on ORF2 capsid protein (**D**) and ORF1b RdRp sequences (**E**). The two panels were independently clustered. All Antarctic strains are highlighted in dark purple. The strain identified in this study is marked in red bold text.

A near-full-length astrovirus genome was also recovered, encoding ORF1a, ORF1b (RdRp), and ORF2 capsid proteins (Figure 4A). Phylogenetic trees were constructed based on the amino-acid sequences of RdRp and the capsid protein, respectively, and analyses were complemented by sequence-identity heatmaps (Figure 4B–E). Phylogenetic analysis firmly placed this isolate within the genus Avastrovirus, which was formally designated “Penguin astrovirus 2025”. Previously reported Antarctic astroviruses were scattered across the tree and failed to form a single monophyletic polar clade. Homology analysis revealed that both the RdRp and ORF2 sequences of this penguin isolate exhibited sequence identities substantially below the accepted astrovirus-species demarcation thresholds (83% for RdRp nucleotides and 75% for the capsid protein) [13], relative to representative astrovirus strains originating from other continents. Accordingly, this isolate is proposed to represent a novel astrovirus species.

### 3.5. Genomic and Phylogenetic Characteristics of Penguin Picornaviridae and Caliciviridae

**Figure 5:**
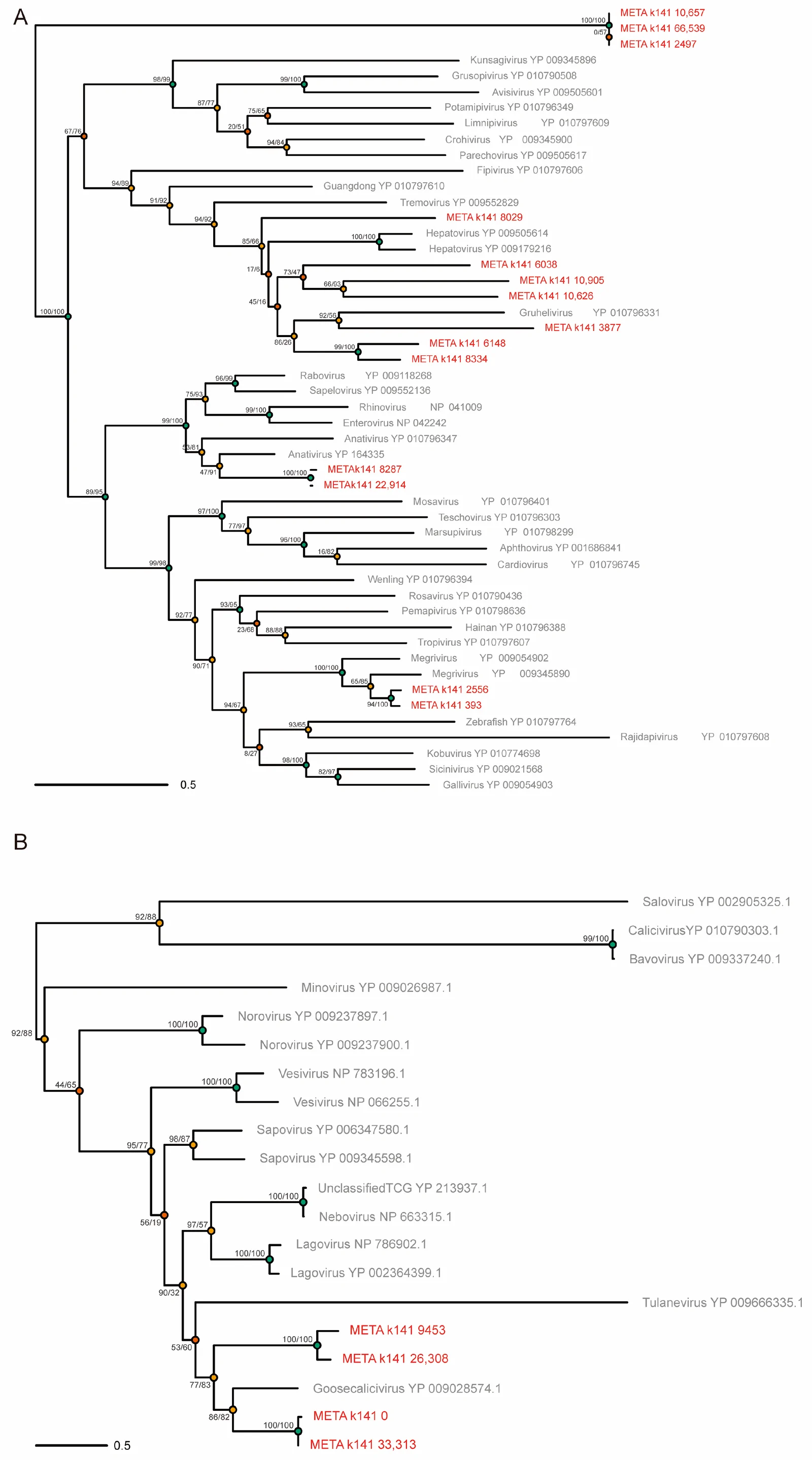
Maximum-likelihood phylogenetic trees constructed based on the amino-acid sequences of viral RdRp proteins. (**A**) Phylogenetic tree for the family *Picornaviridae*; (**B**) Phylogenetic tree for the family *Caliciviridae*. Sequences generated in this study are highlighted in red, and reference sequences retrieved from public databases are shown in grey. Multiple-sequence alignment was performed using MAFFT. Phylogenetic inference was conducted using the maximum-likelihood (ML) method in IQ-TREE with 1000 bootstrap replicates. The lengths of the RdRp amino-acid sequences used for phylogenetic analysis are listed in Table A1. Branch lengths correspond to amino-acid substitutions per site as indicated by the scale bar. “META k141” represents contig identifiers of viral sequences assembled in this study; detailed information for these contigs can be found in Table A1.

Beyond members of the *Coronaviridae* and *Astroviridae*, virome analysis revealed that viruses belonging to the *Picornaviridae* and *Caliciviridae* represented abundant viral fractions in penguin faecal samples (Figure 2B), and multiple viral contigs assigned to these two families were assembled. Maximum-likelihood phylogenetic trees were separately constructed using deduced RdRp amino-acid sequences for *Picornaviridae* and *Caliciviridae*. Sequences assigned to the *Picornaviridae* were dispersed across multiple genera of this family, including Hepatovirus, Megrivirus, and several unclassified picorna-like viruses, reflecting high lineage diversity. Several of these viral clades were closely related to viruses derived from avian, marine-mammal and bat hosts (Figure 5A). Four *Caliciviridae* sequences clustered within the Goosecalicivirus clade and formed two distinct sub-clusters, showing the closest phylogenetic relatedness to goose-derived avian caliciviruses and being clearly separated from other genera within the family *Caliciviridae* (Figure 5B). Major branches received robust nodal bootstrap support, and the scale bar corresponds to an amino-acid genetic distance of 0.5.

## 4. Discussion

This study systematically characterised the faecal viromes of three sympatric Antarctic penguin species, identifying 32 known viral families, with over half of the sequences remaining unclassified. This substantial proportion of unassigned sequences underscores that the gut ecosystems of Antarctic penguins constitute a rich reservoir harbouring considerable, still-undescribed viral diversity. The incompleteness of public viral reference databases, together with short-read transcriptomic data, constrained accurate taxonomic assignment for numerous novel viral contigs. Viral abundance and alpha diversity showed no significant differences among species. Beta diversity analysis indicated that host species significantly influenced viral community composition; however, the low coefficient of determination suggested that host-species density made only a limited contribution to structuring viral communities. Most of the variation in viral communities stemmed from individual characteristics, dietary variation, environmental exposure, and stochastic ecological processes. Dietary-derived algae- and plant-associated viruses were frequently recovered, further illustrating the influence of food resources on gut virome profiles. While highly pathogenic viruses such as H5N1 or Newcastle disease virus were not detected, the possibility of infection cannot be fully excluded given the constrained sampling extent and sample size.

Multiple viral families identified in the faecal viromes of Antarctic penguins, including *Picornaviridae*, *Coronaviridae*, *Astroviridae* and *Caliciviridae,* overlap with common enteric virus groups reported from temperate poultry and other avian hosts [14]. This observation supports the idea that a core set of viral families circulate broadly among Aves across geographically distant regions. Nevertheless, numerous Antarctic viral contigs exhibit substantial sequence divergence from their temperate avian counterparts, indicating local adaptive evolution following viral colonisation of the Antarctic ecosystem. Consistent with previous findings [4], *Picornaviridae* and *Astroviridae* constituted major RNA-viral components in penguin faecal samples. Notably, the deltacoronavirus characterised in the present study represents the first near-complete deltacoronavirus genome obtained from Adélie penguins. Phylogenetic analysis confirms that this virus clusters within previously identified Antarctic seabird deltacoronavirus lineages, supporting the hypothesis that deltacoronaviruses likely experience recurrent cross-species transmission events among sympatric Antarctic seabirds. For the novel astrovirus characterised herein, RdRp- and capsid protein-based phylogenetic constructions revealed a polyphyletic distribution of Antarctic astroviruses. Specifically, Penguin astrovirus 2025 clusters within the avian poultry- and waterfowl-associated Avastrovirus Clade 1, whereas the previously reported Adélie penguin astrovirus (KM587711) belongs to a distinct phylogenetic clade. This pattern suggests multiple independent introductions of astrovirus lineages into Antarctic penguin populations, rather than a single ancient polar-endemic astrovirus ancestor. Homologous recombination is a well-established evolutionary force that shapes the genetic diversity of members within the *Astroviridae*. Given the scope of the present dataset, dedicated recombination detection analyses were not performed. This represents a key limitation for the species demarcation and evolutionary inference in this study. Nevertheless, taxonomic assignment is supported by two independent genetic markers (RdRp and capsid protein), which partially mitigates potential phylogenetic confounding signals derived from recombination events.

Although the detected viruses mainly cause asymptomatic latent infections without current outbreaks, their spillover risks remain non-negligible. The introduction of exotic pathogens can drive adaptive mutations, enhanced virulence, and cross-species transmission. Moreover, high-density breeding colonies and migratory bird movements [15] may accelerate viral spread among Antarctic wildlife, potentially destabilising polar ecosystems. Therefore, establishing long-term systematic pathogen surveillance is therefore essential for tracking viral dynamics and safeguarding polar ecological security.

## Data Availability

All viral nucleotide sequences generated in this study are publicly available in the Science Data Bank (ScienceDB) under the dataset identifier: DOI (https://doi.org/10.57760/sciencedb.0104t), CSTR 31253.11.sciencedb.0104t.

## Abbreviations

The following abbreviations are used in this manuscript:

COI	Cytochrome c oxidase subunit I
TPM	Transcripts Per Million
HPAIV	Highly Pathogenic Avian Influenza Virus
RdRp	RNA-dependent RNA polymerase

## Appendix A

**Table A1:** Metadata of near-full-length viral genomes recovered from penguin faecal samples in this study.

Sample ID (Red Label)	Assembly Length (bp)	Viral Family	Species	Host	The Length of Each Fragment Used for Phylogenetic Analysis
META k141 10657	4066	*Picornaviridae*	Fur seal picorna-like virus	Pygoscelis antarcticus	RdRp 345 aa
META k141 66539	8774	*Picornaviridae*	*Labidiaster annulatus*-associated virus	Pygoscelis antarcticus	RdRp 398 aa
META k141 2497	4707	*Picornaviridae*	Fur seal picorna-like virus	Pygoscelis antarcticus	RdRp 345 aa
META k141 8029	7243	*Picornaviridae*	Whistler virus	Pygoscelis antarcticus	RdRp 519 aa
META k141 6038	6311	*Picornaviridae*	hepatovirus H2	Pygoscelis antarcticus	RdRp 522 aa
META k141 10905	7661	*Picornaviridae*	Loch Leven virus	Pygoscelis adeliae	RdRp 523 aa
META k141 10626	7648	*Picornaviridae*	Mute swan faeces-associated hepatovirus	Pygoscelis antarcticus	RdRp 524 aa
META k141 3877	7540	*Picornaviridae*	Waterwitch virus	Pygoscelis adeliae	RdRp 516 aa
META k141 6148	7473	*Picornaviridae*	Sphenimaju virus	Pygoscelis papua	RdRp 519 aa
META k141 8334	7404	*Picornaviridae*	Sphenimaju virus	Pygoscelis papua	RdRp 520 aa
META k141 8287	7975	*Picornaviridae*	Bat picornavirus	Pygoscelis papua	RdRp 482 aa
META k141 22914	7859	*Picornaviridae*	Bat picornavirus	Pygoscelis papua	RdRp 483 aa
META k141 2556	9669	*Picornaviridae*	megrivirus E1	Pygoscelis antarcticus	RdRp 502 aa
META k141 393	9714	*Picornaviridae*	megrivirus E1	Pygoscelis adeliae	RdRp 502 aa
META k141 9453	8232	*Caliciviridae*	Goose calicivirus	Pygoscelis papua	RdRp 516 aa
META k141 26308	8324	*Caliciviridae*	Goose calicivirus	Pygoscelis adeliae	RdRp 539 aa
META k141 0	8423	*Caliciviridae*	*Caliciviridae* sp.	Pygoscelis adeliae	RdRp 542 aa
META k141 33313	8357	*Caliciviridae*	*Caliciviridae* sp.	Pygoscelis adeliae	RdRp 543 aa
META k141_13325	6919	*Astroviridae*	astroviruses	Pygoscelis antarcticus	RdRp 480 aa/1440 nt; Capsid Protein 683 aa/2049 nt
META k141_32723	26196	*Coronaviridae*	Adeliae penguin deltacoronavirus	Pygoscelis adeliae	Genome 23,506 nt RdRp 924 aa/2772 nt
META k141_6555	8114	/	weddell virus	Pygoscelis antarcticus	/
META k141_26019	8098	/	weddell virus	Pygoscelis antarcticus	/
